# Mitochondrial dynamics and diabetic kidney disease: Missing pieces for the puzzle of therapeutic approaches

**DOI:** 10.1111/jcmm.17116

**Published:** 2021-12-09

**Authors:** Phoom Narongkiatikhun, Siriporn C. Chattipakorn, Nipon Chattipakorn

**Affiliations:** ^1^ Division of Nephrology Department of Internal Medicine Faculty of Medicine Chiang Mai University Chiang Mai Thailand; ^2^ Cardiac Electrophysiology Research and Training Center Faculty of Medicine Chiang Mai University Chiang Mai Thailand; ^3^ Center of Excellence in Cardiac Electrophysiology Research Chiang Mai University Chiang Mai Thailand; ^4^ Cardiac Electrophysiology Unit Department of Physiology Faculty of Medicine Chiang Mai University Chiang Mai Thailand

**Keywords:** diabetic kidney disease, mitochondrial dynamics, mitochondrial functions, pathogenesis, pharmacological interventions

## Abstract

Diabetic kidney disease (DKD) is a common microvascular complication among diabetic patients. Once the DKD has developed, most of the patients inevitably progress to the end‐stage renal disease (ESRD). Although many new therapeutic strategies have attempted to demolish the root of the pathogenesis of DKD, the residual risks of ESRD still remained. Alteration of mitochondrial dynamics towards mitochondrial fission concurrent with the mitochondrial dysfunction is the characteristic that is usually seen in various diseases, including DKD. It has been proposed that those perturbation and their cooperative networks could be responsible for the residual risk of ESRD in DKD patients. In this review, the collective evidence of alteration in mitochondrial dynamics and their associations with the mitochondrial function from *in vitro*, *in vivo* and clinical reports of DKD are comprehensively summarized and discussed. In addition, both basic and clinical reports regarding the pharmacological interventions that showed an impact on the mitochondrial dynamics, and the correlation with the renal parameters in DKD is presented. Understanding these complex mechanisms in combination with the existing therapeutic modalities could bring a new opportunity to overcome the unresolvable problem of DKD.

## 1 INTRODUCTION

Diabetic kidney disease (DKD) is the most common cause of chronic kidney disease (CKD), and the major aetiology of end‐stage renal disease (ESRD), contributing to renal replacement therapy worldwide.^1^ The presence of DKD among diabetic patients is associated with higher morbidity and mortality, comparing to other vascular complications.^2^ In diabetes mellitus (DM), substantial comorbid diseases such as hypertension and dyslipidaemia, which are the common illness that often found in the same patient, can enhance the deterioration of DKD.^3^ Nevertheless, recent data demonstrate that although the incidence of DKD is stabilized, this is due to improved treatment on those comorbidities, not DKD.^4^

The pathogenesis of DKD consists of two main mechanisms including haemodynamic and metabolic pathways.^3^ Molecular studies demonstrated that each pathway involved numerous complicated vital substrates that are responsible for the development of DKD. Although many clinical studies using emerging therapies and possible beneficial strategies intended to reverse those pathological keys, none of those succeeded in stopping the disease progression.^4^, ^5^ It has been proposed that once the kidney involvement is observed, the progression to ESRD is inevitable overtime.^6^ According to those facts, understanding the molecular mechanisms responsible for the development of DKD is crucial for developing strategies to combat with DKD and ESRD. Recently, the roles of mitochondria and mitochondrial dynamics have been extensively investigated and have been proposed as one of the jigsaw puzzles to complete the mechanistic picture in DKD.^7^, ^8^

Mitochondria are membrane‐bound organelles which are known as the power generator of every cell. They incessantly alter their size, shape and number through the process of ‘mitochondrial dynamics’, which consists of mitochondrial fusion and fission, in response to cellular energy requirement and to maintain their homeostasis.^9^ Mitochondrial fusion causes the mitochondria joining together for sharing the substrates between injured mitochondria to eliminate the damaged components and recover overall mitochondrial functions.^10^ However, mitochondrial fission is activated if the cellular damage is occurred, and the broken fragments will be eliminated through the budding off vesicle which is sent to the recycling process, an event known as mitophagy.^11^ Simultaneously, mitochondrial biogenesis is a process which promotes mitochondrial DNA duplication, and to synthesize revitalize organelles substituting the dysfunctional mitochondria.^12^ Under physiological condition, the balance between fusion and fission must be maintained.^13^ Growing evidence demonstrates that an alteration in mitochondrial dynamics is associated with the underlying pathogenesis of various diseases including malignancy, cardiovascular disease and neurodegenerative disease.^14^, ^15^, ^16^, ^17^ DKD has also been shown to involve in this mitochondrial networking abnormality.^18^ Interestingly, these phenomena seem to occur preceding the development of both clinical and laboratory abnormalities in DKD.^7^, ^19^ Since impaired balance of mitochondrial dynamics could be responsible for DKD pathogenesis, the modulation on those processes to restore its homeostasis might be the therapeutic strategies to correct the unresolvable problem of DKD. Despite this proposed hypothesis, the current evidence from both basic and clinical reports is still limited regarding the precise mechanism of mitochondrial dynamics on DKD and the effective interventions targeting mitochondrial dynamics.

This review intends to summarize the evidence which is available from the *in vitro*, *in vivo* and clinical studies regarding the roles of mitochondrial dynamics on DKD. It also focuses on both mechanistic and interventional aspects, concurrently with the discussion of their potential application in the clinical practice in DKD patients. Lastly, this review aims to encourage more basic and clinical investigations on the roles of mitochondrial dynamics in DKD to complete the jigsaw puzzles on our understanding of DKD mechanisms and to pave ways for better therapeutic strategies in those patients.

The PubMed database was used for the search for literatures published in English language before October 2020. Diabetic kidney disease and mitochondrial dynamics were used as keywords. All the results from the search were reviewed, and the relevant articles were identified for further reviewed. The pertinent findings of each literature were extracted and summarized in this review.

## 2 NATURAL HISTORY OF DIABETIC KIDNEY DISEASE AND ITS CORRELATION WITH MITOCHONDRIAL DYNAMICS

Diabetic kidney disease is one of the microvascular complications among diabetic patients.^20^ Typical laboratory manifestations are the presence of impaired kidney function, concurrent with progressive increment of albuminuria, which are concordant with the duration of being diagnosed DM.^20^ DKD consists of five stages including hyperfiltration, silence, incipient, overt nephropathy and ESRD.^20^ The higher the stage of DKD, the worse of the glomerular filtration rate (GFR), the degree of albuminuria and the subsequent development of hypertension are to be found.^20^ According to the natural history of DKD, hyperglycaemia is believed to be the central initiator of the disease.^21^ Nonetheless, tight glycaemic control strategy was failed to demonstrate the effectiveness on the ESRD prevention,^22^, ^23^, ^24^, ^25^, ^26^ and even worse with the possible harm in term of increasing mortality.^24^ In addition, recent clinical studies reported the beneficial effect of sodium‐glucose cotransporter 2 inhibitors (SGLT‐2i), which is one of the glucose‐lowering agent categories, on retarding DKD progression as a result beyond glucose‐lowering effect.^27^ All of these findings suggested that there are still undiscovering mechanisms for the pathogenesis of DKD.

Previous findings revealed that longstanding hyperglycaemic state generated intracellular oxidative stress accompanied with the disruption of mitochondrial dynamics by promoting mitochondrial fission^28^ and suppressing mitochondrial fusion.^7^ These alterations also impaired biogenesis and mitophagy, leading to increased mitochondrial fragmentation, enhanced mitochondria permeability transition pore (mPTP) opening, induced cytochrome C leakage into cytosol, and finally stimulated the cellular apoptosis.^7^ Furthermore, mitochondrial fission was also found to be associated with the worsening of kidney parameters such as GFR and albuminuria in DKD.^29^, ^30^ Taken together, these findings accentuated the vital role of mitochondrial dynamics on the pathogenesis of DKD, and that it can be the potential modulated pathway that might lead to the novel targeted therapy for DKD.

In molecular studies, mitochondrial fusion is reflected through the presence of mitofusin 1 (MFN1), mitofusin 2 (MFN2) and optic atrophy protein 1 (OPA1).^31^ MFN1 and MFN2 are located at the outer membrane of the mitochondria, while OPA1 is situated at the inner membrane. Interaction between MFN and OPA1 from each organelle proceeds the fusion process, which causes less mitochondrial fragmentation, and more elongated mitochondria as describe by increasing of aspect ratio (AR).^32^, ^33^ Mitochondrial fission is evaluated from the expression of dynamin‐related protein 1 (DRP1), mitochondrial fission protein 1 (Fis1) and mitochondrial fission factor (MFF).^33^ DRP1 is a main cytosolic fission effector that once activated, it will translocate to the outer membrane of mitochondria and adhere with MFF and Fis1 guided for forming a separation ring‐like structure.^32^ On the contrary to fusion, fission causes more mitochondrial fragmentation and mitochondrial length shortening as demonstrate by decreasing in AR. In biogenesis activity, it is detected from the expression of peroxisome proliferator‐activated receptor‐gamma coactivator 1‐alpha (PGC‐1α).^34^ For mitophagy, PTEN‐induced putative kinase protein 1 (PINK1) and E3 ubiquitin‐protein ligase parkin (Parkin) are the two important proteins for this process.^31^ Both representatives are sequentially activated and tagged the injured mitochondria which had lost their membrane potential, in order to trigger the autophagosome formation.^31^ After that, enzymes are released into the autophagosome to eliminate the content inside which subsequently either excreted or reutilized.^35^ Interestingly, many new molecular markers related to mitochondrial dynamics and their co‐operated networks in DKD have been identified in *in vitro*, *in vivo* and clinical studies.

## 3 MITOCHONDRIAL DYNAMICS ALTERATIONS IN DIABETIC KIDNEY DISEASE: EVIDENCE FROM *IN VITRO* AND *IN VIVO* STUDIES

*In vitro* study of the kidney cells under diabetic milieu demonstrated that the renal mitochondrial dynamics were altered as indicated by increased mitochondrial fission, while the mitochondrial fusion was attenuated.^13^, ^28^, ^36^, ^37^, ^38^, ^39^, ^40^ Furthermore, the mitochondrial biogenesis was suppressed,^36^, ^39^ and the mitophagy was impaired.^13^, ^40^ All of these changes resulted in mitochondrial dysfunction as demonstrated by the shortening of mitochondrial length, mitochondrial depolarization, increased oxidative stress generation and decreased adenosine triphosphate (ATP) production.^13^, ^28^, ^36^, ^37^, ^38^, ^39^, ^40^

**FIGURE 1:**
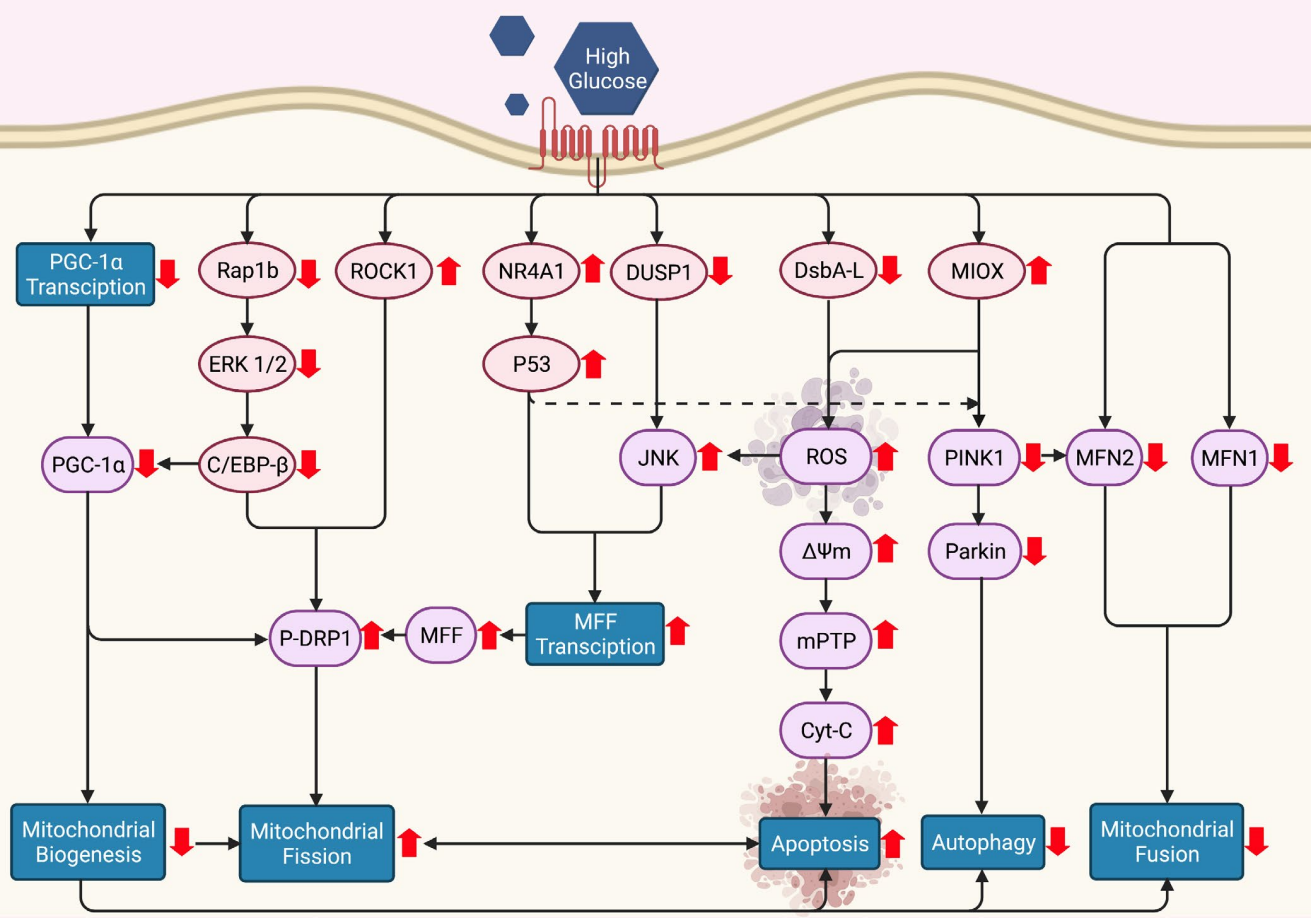
Regulatory signalling of mitochondrial dynamics and their cooperative networks under diabetic milieu. Excess fission was mediated through the upstream signalling that directly stimulated DRP1 phosphorylation and DRP1 facilitated proteins. These included Rap1b ‐ ERK1/2 – C/EBP‐ β cascade, ROCK1, NR4A1 – P53 and DUSP1 – JNK pathway. In addition, fission could indirectly activate through the excess of ROS generation and apoptosis as a result of decreased DsbA‐L and increased of MIOX. The downregulation of mitochondrial fusion was regulated through the MIOX, which intervened the PINK1 facilitated Parkin and MFN2 interactions, and the direct inhibitory effect of high glucose state. Mitochondrial biogenesis was mainly suppressed via the decreased of PGC‐1α in consequence of direct high glucose mediated, and the alteration of Rap1b ‐ ERK1/2 – C/EBP‐ β pathway. Autophagy (mitophagy) was impaired through the perturbation of NR4A1‐P53 and MIOX – PINK1 – Parkin signalling. Notably, alteration of each process was conversely deteriorated one another. C/EBP‐ β, CCAAT‐enhancer binding protein; Cyt‐C, cytochrome C; DsbA‐L, Disulfide‐bond A oxidoreductase‐like protein; DUSP1, Dual‐specificity protein phosphatase 1; ERK1/2, extracellular signal‐related kinase 1/2; JNK, c‐Jun N‐ terminal kinase; MFF, mitochondrial fission factor; MFN 1, mitofusin 1; MFN 2, mitofusin 2; MIOX, myo‐inositol oxygenase; mPTP, mitochondrial permeability transition pore; PDrp1, phosphorylated dynamin‐related protein 1; NR4A1, nuclear receptor subfamily 4 group A member 1; PGC‐1α, peroxisome proliferator‐activated receptor‐gamma coactivator 1‐alpha; Pink1, PTEN‐induced putative kinase 1; Rap1b, Ras‐ proximate 1b; ROCK1, Rho‐associated coiled coil‐containing protein kinase 1; ROS, reactive oxygen species; ΔΨm, mitochondrial membrane potential

The regulatory pathways that mediated the mitochondrial fission under high glucose exposure composed of many proteins (Figure 1), including Rho‐associated coiled coil‐containing protein kinase 1 (ROCK1),^28^ Ras‐proximate 1b (Rap1b),^36^ Disulphide‐bond A oxidoreductase‐like protein (DsbA‐L),^37^ Dual‐specificity protein phosphatase 1 (DUSP1),^38^ PGC‐1α,^39^ myo‐inositol oxygenase (MIOX)^13^ and the nuclear receptor subfamily 4 group A member 1 (NR4A1).^40^ Some of these proteins activated the fission process through increasing transcription or phosphorylation of the fission substrates, such as DRP1 and MFF, while the others promoted mitochondrial fission via activation of ROS.^13^, ^37^

Unlike mitochondrial fission, the underlying signal that was responsible for the decreasing of mitochondrial fusion among diabetic milieu was not apparently defined. Most *in vitro* studies reported only the reduction of fusion activity without demonstrated the regulatory pathway.^36^, ^38^, ^40^ However, there is one study proposed that fusion was attenuated through the increasing of MIOX, which then suppressed the PINK1 and led to the reduction of Parkin—MFN2 interactions.^13^ For mitochondrial biogenesis and mitophagy, each process was demonstrated to be suppressed via the cascade that involved in reducing the expression of PGC‐1α, and PINK1‐Parkin, respectively.^13^, ^36^, ^39^, ^40^ A summary of findings from those *in vitro* reports is shown in Table 1.

**TABLE 1:** Mitochondrial dynamics alterations in hyperglycaemic kidney cells

Model	Major findings									Interpretation	Ref
	Mitochondrial dynamics		Mitochondrial function				Apoptosis	Autophagy	Biogenesis		
	Fusion	Fission	mtMo	ΔΨm	Oxidative stress	ATP					
**Cultured mouse podocytes & kidney mECs**											
HG	–	↑ mtDrp1 ↑ pDrp1 Ser600	↓ AR (P, E)	–	mt ROS	–	↑ AnnexinV ↑ Bax ↑ Cyt‐C ↑ Caspase3 activity	–	–	High glucose‐induced mitochondrial fission through an activation of ROCK1.	28
si‐ROCK1 + HG	–	↓ mt fragment ↓ mt Drp1	↑ AR (P, E)	–	–	–	↓ AnnexinV ↓ Bax ↓ Cyt‐C ↓ Caspase3 activity	–	–		
cA‐ROCK1 transfected podocyte	–	↑ mt fragment ↑ mt Drp1	↓ AR (P, E)	–	–	–	↑ Bax ↑ Cyt‐C ↑ Caspase3 activity	–	–		
cA‐ROCK1 transfected podocyte + shDrp1 vs. HG	–	↓ mt fragment ↓ mt Drp1	↑ AR (P, E)	–	↓ mt ROS	–	↓ AnnexinV	–	–		
Flag‐ Drp1 (S600A) + Drp1 shRNA + HG	–	–	↑ AR (P, E)	–	–	–	↓ AnnexinV	–	–		
**HK‐2 cells**											
HG	↓ MFN2	↑ pDrp1 Ser637 ↑ mt fragment ↓ Rap1b activity	–	↓	↑ Intra‐cellular ROS ↑ mtROS	–	↑ mtDNA fragment ↑ Apoptotic cell ↑ Cyt‐C ↑ Cleaved Caspase3	–	↓ mRNA PGC‐1α (dose dependent) ↓ PGC‐1α protein expression (dose dependent) ↓ C/EBP‐β	High glucose impaired mitochondrial dynamics through interfering Rap1b – ERK1/2 – C/EBP‐β – PGC‐1α signalling pathway.	36
Rap1bG12V (Rap1 overexpression) + HG	↑ MFN2	↓ pDrp1 Ser637 ↓ mt fragment	–	↑	↓ Intra‐cellular ROS ↓ mtROS	–	↓ mtDNA fragment ↓ Apoptotic cell ↓ Cyt‐C ↓ Cleaved Caspase3	–	↑ PGC‐1α promoter activity ↑ mRNA PGC‐1α ↑ PGC‐1α protein expression ↑ C/EBP‐β		
C/EBP‐β siRNA + Rap1bG12V + HG	↓ MFN2	↑ pDrp1 Ser637	–	↓	↑ Intra‐cellular ROS ↑ mtROS	–	↑ mtDNA fragment ↑ Apoptotic cell ↑ Cyt‐C ↑ Cleaved Caspase3	–	↓ mRNA PGC‐1α ↓ PGC‐1α protein expression		
ERK 1/2 siRNA + Rap1bG12V + HG	–	–	–	–	–	–	–	–	↓ mRNA PGC‐1α ↓ PGC‐1α protein expression ↓ C/EBP‐β		
CEBP‐β siRNA + ERK ½ siRNA + Rap1bG12V + HG	↓ MFN2	↑ pDrp1 Ser637	–	–	–	–	–	–	↓ mRNA PGC‐1α ↓ PGC‐1α protein expression		
PGC‐1α siRNA + Rap1bG12V + HG	–	–	–	–	↑ Intra‐cellular ROS ↑ mtROS	–	↑ mtDNA fragment ↑ Apoptotic cell ↑ Cyt‐C ↑ Cleaved Caspase3	–	–		
PGC‐1α siRNA + CEBP‐β siRNA + Rap1bG12V + HG	–	–	–	↓	–	–	↑ mtDNA fragment	–	–		
Rap1bS17N (mutant Rap1) + HG vs. Rap1G12V + HG	↓ MFN2	↑ pDrp1 Ser637 ↑ mt fragment	–	–	–	–	↑ Apoptotic cell	–	–		
**HK‐2 cells**											
HG	–	↑ mt fragment ↑ p‐JNK ↑ p‐MFF ↑ MFF mRNA	↓ AR (T)	–	↑ mtROS ↓ DsbA‐L	–	–	–	–	High glucose attenuated DsbA‐L, leading to increased mitochondrial ROS generation, JNK activation, MFF transcription, and mitochondrial fission.	37
siDsbA‐L + HG	–	↑ mt fragment ↑ p‐JNK ↑ p‐MFF ↑ MFF mRNA	↓ AR (T)	–	↑ mtROS ↓ DsbA‐L	–	–	–	–		
DsbA‐L O/E + HG	–	↓ mt fragment	↑ AR (T)	–	↓ mtROS ↑ DsbA‐L	–	–	–	–		
mitoQ (anti‐mtROS) + siDsbA‐L + HG vs. HG	–	↓ mt fragment ↓ p‐JNK	↑ AR (T)	–	↓ mt ROS	–	–	–	–		
SP (JNK inhibitor) + siDsbA‐L + HG vs. HG	–	↓ mt fragment ↓ p‐JNK ↓ p‐MFF ↓ MFF mRNA	↑ AR (T)	–	–	–	–	–	–		
**Human renal mesangial cells transfected with Adenovirus with DUSP1 overexpression**											
HG	↑ MFN1 ↑ OPA1	↓ Drp1 ↓ p‐MFF ↓ p‐JNK	↑ AR (M)	↑	↓ mt ROS	–	↓ Caspase3 expression ↓ Caspase9 expression ↓ mPTP ↓ Cyt‐C ↓ Bax ↑ Bcl‐2 ↑ c‐IAP1 ↓ LDH release ↑ Cell viability	–	–	High glucose‐stimulated mitochondrial fission dependently on DUSP1.	38
FCCP pretreatment (activate mt fission) + DUSP1 overexpression + HG	–	–	–	–	–	–	↑ Caspase9 ↑ LDH release	–	–		
Mutant MFF (S146 replaced with aspartic acid) + DUSP1 overexpression + HG	–	↑ mt fragment ↑ p‐MFF	↓ AR (M)	–	–	–	↓ Cell viability	–	–		
JNK pathway activator (Ani) + HG	–	↑ p‐JNK ↑ p‐MFF	–	–	↑ mt ROS	↓	↑ TUNEL ↑ Caspase3 activity	–	–		
**Human renal mesangial cells**											
JNK pathway inhibitor (SP) + HG	–	↓ p‐MFF ↓ p‐JNK	–	–	↓ mt ROS	↑	↓ TUNEL ↓ Caspase3 activity	–	–		38
**Rat glomeruli mesangial cells**											
HG	–	↑ mt fragment ↑ Drp1	↓ AR (M) ↓ FF (M)	–	–	–	–	–	↓ PGC‐1α mRNA ↓ PGC‐1α protein expression	PGC‐1α attenuated mitochondrial fission via decreased Drp1.	39
pcDNA3‐PGC‐1α + HG	–	↓ mt fragment ↓ Drp1	↑ AR (M) ↑ FF (M)	–	↓ mt ROS	–	–	–	↑ PGC‐1α		
Drp1 + pcDNA3‐PGC‐1α + HG	–	↑ mt fragment	↓ AR (M) ↓ FF (M)	–	↑ mt ROS	–	–	–	–		
**HK‐2 cells and LLC‐PK1 (porcine)**											
HG	↓ MFN2 ↔ OPA1	↑ mt fragment ↑ Drp1 ↑ FIS1	–	–	↑ mtROS ↑ MIOX	–	↑ Bax ↑ Cyt‐C	↓ LC3 ↔ Atg5 ↓ Pink 1 ↓ Parkin	–	High glucose‐stimulated MIOX which subsequently activated mitochondrial fission, and impaired autophagy through ROS activation, and Pink 1 inhibition, respectively.	13
**Human renal mesangial cells**											
si‐NR4A1 + HG	↑ MFN1 expression ↑ OPA1 expression	↓ mt Drp1 expression ↓ MFF transcription ↓ MFF expression ↓ p‐p53 expression	↑ AR (M)	↑	–	↑	↓ Nuclear expression of Cyt‐C ↓ Caspase3 expression ↓ Caspase9 expression ↓ Caspase 9 activity ↓ Bax‐expression ↓ LDH release ↓ mPTP opening rate ↑ Bcl‐2 expression ↑ c‐IAP1 expression	↑ Parkin transcription ↑ mt Parkin expression ↑ LC3‐II/LC3‐I ↓ p62 expression ↑ mt LC3‐II ↓ Tom20 expression ↓ Tim23 expression ↑ No. of mitophagy	–	NR4A1 – p53 signalling was activated under diabetic milieu, which accentuated mitochondrial fission and suppressed mitophagy.	40
si‐p53 + HG	–	↓ MFF transcription ↓ MFF expression ↓ p‐p53 expression	↑ AR (M)	–	↓ ROS	–	↓ TUNEL	↑ Parkin transcription ↑ mt Parkin expression ↑ LC3‐II/LC3‐I ↓ p62 expression ↓ Tom20 expression ↓ Tim23 expression	–		

Abbreviations: AR, aspect ratio; Atg5, autophagy‐related gene 5; Bax, Bcl‐2 associated X protein; Bcl‐2, B‐cell lymphoma 2; C/EBP‐ β, CCAAT‐enhancer binding protein; c‐IAP1, Cellular Inhibitor of Apoptosis Protein 1; Cyt‐C, cytochrome C; Drp1, dynamin‐related protein 1; DsbA‐L, Disulphide‐bond A oxidoreductase‐like protein; DUSP1, Dual‐specificity protein phosphatase 1; E, endothelial cell; ERK1/2, extracellular signal–related kinase 1/2; FCCP, Carbonyl cyanide‐p‐trifluoromethoxyphenylhydrazone; FF, form factor; FIS1, fission 1 protein; GBM, glomerular basement membrane; HG, high glucose; JNK, c‐Jun N‐ terminal kinase; LC3, light chain 3; LDH, lactate dehydrogenase; M, mesangial cell; mEC, microvascular endothelial cells; MFF, mitochondrial fission factor; MFN, mitofusin; MIOX, myo‐inositol oxygenase; Mo, morphology; mPTP, mitochondrial permeability transition pore; mRNA, messenger ribonucleic acid; mt, mitochondria; mt, mitochondria; NG, normal glucose; NR4A1, nuclear receptor subfamily 4 group A member 1; O/E, overexpression; OPA1, optic atrophy 1; P, podocyte; PGC‐1α, peroxisome proliferator‐activated receptor‐gamma coactivator 1‐alpha; Pink1, PTEN‐induced putative kinase 1; Rap1b, Ras‐ proximate 1b; ROCK1, Rho‐associated coiled coil‐containing protein kinase 1; ROS, reactive oxygen species; si, silencing; T, tubular cell; TIM23, translocase of the inner membrane 23; TOM20, translocase of the outer membrane 20; TUNEL; terminal deoxynucleotidyl transferase dUTP nick end labelling; WCL, whole cell lysate.

**FIGURE 2:**
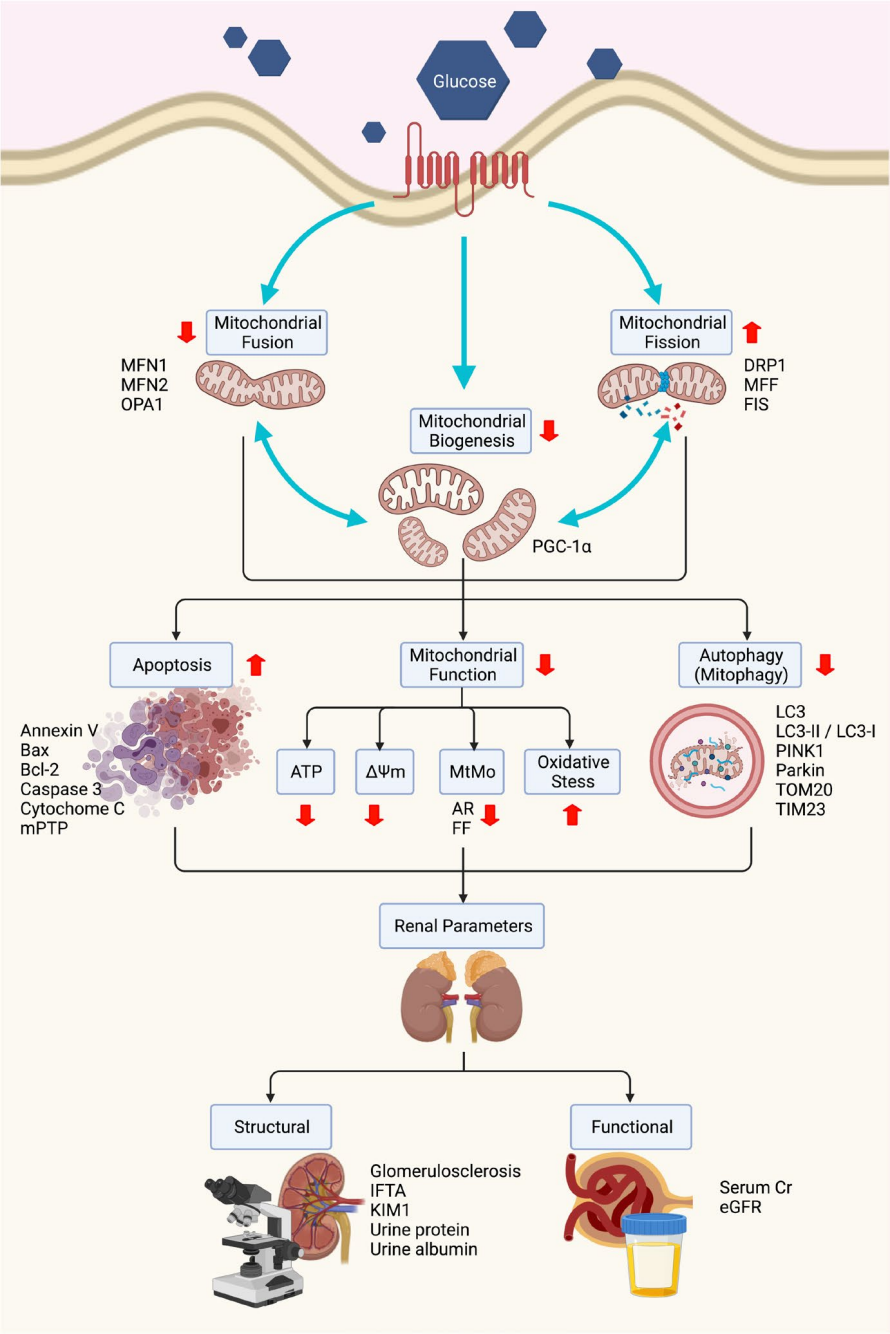
Association between an alteration of mitochondrial dynamics and other cooperative networks with the laboratory parameters of diabetic kidney disease. High glucose state initially altered mitochondrial dynamics by shifting homeostasis towards fission, while mitochondrial fusion was attenuated. Concurrently, mitochondrial biogenesis was downregulated. Each perturbation conversely insulted on one another, which leaded to the development of vicious cycle. As a result of those changes, cellular apoptosis, mitochondrial dysfunction and decreased of mitophagy were subsequently occurred. Those complex interconnected processes were associated with the worsening of renal parameters and were considered as the underlying pathogenesis of diabetic kidney disease. AR, aspect ratio; ATP, adenosine triphosphate; Bax, Bcl‐2 associated X protein; Bcl‐2, B‐cell lymphoma 2; Cr, creatinine; Drp1, dynamic‐related protein 1; eGFR, estimated glomerular filtration rate; FF, form factor; FIS1, fission 1 protein; LC3, light chain 3; IFTA; interstitial fibrosis and tubular atrophy; KIM‐1, kidney injury molecule‐1; MFF, mitochondrial fission factor; MFN 1, mitofusin 1; MFN 2, mitofusin 2; mPTP, mitochondrial permeability transition pore; mtMo, mitochondrial morphology; OPA1, optic atrophy 1; PGC‐1α, peroxisome proliferator‐activated receptor‐gamma coactivator 1‐alpha; Pink1, PTEN‐induced putative kinase 1; TIM23, translocase of the inner membrane 23; TOM20, translocase of the outer membrane 20; ∆Ψm, mitochondrial membrane potential

Currently, whether the perturbation of mitochondrial dynamics occurred prior to the renal structure and the laboratory chemistry abnormalities are still debated.^7^ The mapping‐time course of mitochondrial dynamics in the kidney among diabetic rat models revealed that at the early phase, mitochondrial fusion and biogenesis were increased as the compensatory mechanism to the excessive mitochondrial fission, which was augmented by the hyperglycaemia.^7^ Unfortunately, mitochondrial fission was predominant as demonstrated by an increasing of mitochondrial fragmentation, reducing of the AR, antioxidant and the mitochondrial bioenergetics. For the natural history of DKD, serum cystatin C was initially decreased, which related to the hyperfiltration stage, whereas the surrogate markers of renal injury and renal dysfunction were unremarkably changed.^7^ With the persistence of hyperglycaemia, mitochondrial dynamics were shifting towards fission, while fusion was decreased. At this time, there were evidence of glomerulosclerosis, progressive increasing of urinary albumin excretion and urinary kidney injury molecule‐1 (KIM‐1).^7^ All of these findings from *in vivo* studies indicated that alterations of renal mitochondrial dynamics occurred prior to the development of abnormal histology and laboratory parameters related to DKD. The association between an alteration of mitochondrial dynamics and other cooperative networks with the laboratory parameters of diabetic kidney disease is illustrated in Figure 2.

*In vivo* studies also provided additional association of mitochondrial dynamics with the laboratory parameters related to DKD. Disturbance of renal mitochondrial dynamics contributed to the abnormal kidney histology and renal parameters.^7^, ^28^, ^32^, ^36^, ^37^, ^41^, ^42^ Notably, genetically modification on mitochondrial associated proteins which involved in the regulatory signalling pathway could alleviate the deteriorating effect of DKD.^28^, ^32^, ^36^, ^37^, ^41^ All of these findings suggested that early modulation on renal mitochondrial dynamics to restore their balance before the structural and functional changes are observed could be the solution not only preventing the disease progression but also eliminating the risk of ESRD. A summary of the findings from *in vivo* reports is shown in Table 2.

**TABLE 2:** Mitochondrial dynamics alterations in diabetic kidney disease: reports from *in vivo* studies

Model	Major findings								Histology	Renal parameters	Interpretation	Ref
	Mitochondrial dynamics		Mitochondrial function					Apoptosis				
	Fusion	Fission	mtMo	ΔΨm	Oxidative stress	ATP	OCR					
**STZ‐induced diabetes rats**												
	↑ OPA1‐L1 (4 weeks) ↓ OPA1‐L1 (32 weeks) ↑ OPA1‐L2 (4 weeks) ↑ MFN1 mRNA (4 weeks) ↑ MFN2 mRNA (4 weeks)	↑ mt fragment (4, 32 weeks) ↑ OPA1‐S2 (16 weeks) ↑ OPA1‐S3 (32 weeks) ↑ MFF mRNA (4 weeks)	↓ AR (T) (4, 32 weeks)	–	↑ H2O2 (8, 16, 32 weeks) ↓ Mn‐SOD (4, 8, 16, 32 weeks)	↓ (4,8, 32 weeks)	↑ (32 weeks)	↑ mPTP (8, 16, 32 weeks)	↑ Glomerulo‐sclerosis (8, 16, 32 weeks)	↓ Serum Cystatin C (4, 8, 16, 32 weeks) ↑ UAE (16, 32 weeks) ↑ Urine KIM‐1 (32 weeks)	Mitochondrial dynamics and bioenergetics were altered before the significant development of abnormal renal parameters related to diabetic kidney disease.	7
**STZ‐induced diabetes rats**												
	–	↑ pDrp1	↓ AR (T)	↓	↑ mtROS ↑ H2O2	–	–	↑ TUNEL ↑ mPTP opening ↓ Pro‐Caspase3 ↓ Pro‐Caspase9 ↑ Cleaved Caspase3 ↑ Cyt‐C	↑ Col I ↑ Fn ↑ mm	↑ UAE ↑ UGGT ↑ Urine β–NAG	Rap1b was downregulated in diabetic rats and was associated with increased mitochondrial fission, oxidative stress, cellular apoptosis and worsen renal parameters.	36
Rap1bG12V	–	↓ pDrp1	↑ AR (T)	↑	↓ mtROS ↓ H2O_2_	–	–	↓ TUNEL ↓ mPTP opening ↑ Pro‐Caspase3 ↑ Pro‐Caspase9 ↓ Cleaved Caspase3 ↓ Cyt‐C	↓ Col I ↓ Fn ↓ mm	↓ UAE ↓ UGGT ↓ Urine β‐NAG		
**STZ‐induced diabetes mice**												
	↓ MFN1 ↓ MFN2	↑ mt fragment ↑ Drp1 ↑ FIS1 ↑ p‐ MFF ↑ p‐JNK	↓ AR (T)	–	↑ mtROS ↓ DsbA‐L	–	–	↑ TUNEL	↑ Tubular disruption ↑ Enlarged glomeruli ↑ mm ↑ IFTA	↑ Urine β‐NAG ↑ KIM‐1	DsbA‐L was reduced in diabetic mice, which contributed to ROS generation, increased mitochondrial fission, and reduced mitochondrial fusion.	37
DsbA‐L−/−	↔ MFN1 ↓ MFN2	↑ mt fragment ↑ Drp1 ↔ FIS1 ↑ p‐MFF ↑ p‐JNK	↓ AR (T)	–	↑ mtROS ↓ DsbA‐L	–	–	↑ TUNEL	↑ Tubular disruption ↑ Enlarged glomeruli ↑ mm ↑ IFTA	↑ Urine β‐NAG ↑ KIM‐1		
**STZ‐induced diabetes fed with high‐fat diet‐fed rats**												
	–	–	–	–	↑ mtROS ↑ 8‐OhdG	–	–	–	↑ Enlarged glomeruli ↑ Fn score	↑ SCr ↑ 24‐hr mALB	PGC‐1α was decreased in diabetic rats and was associated with worsening of renal function.	39
**STZ‐induced diabetes mice**												
ROCK1 KO	–	↓ mt fragment ↓ mtDrp1	AR (P)	–	↓ mtROS	–	–	↓ Frequency of apoptosis ↓ Caspase3 activity ↓ Bax ↓ Cyt‐C	↓ mm ↓ GBM thickness ↑ P No.	↓ UACR	Diabetes increased ROCK1, and genetically removal of ROCK1 alleviated kidney injury.	28
**Podocin‐Specific cA‐ROCK1 transgenic (overexpression) mice**												
	–	–	–	–	–	–	–	–	↑ mm ↑ GBM thickness ↑ P No.	↑ UACR		28
**STZ‐induced diabetes mice**												
Podocin‐Specific cA‐ROCK1 transgenic (overexpression)	–	↑ mt fragment ↑ mt Drp1	↓ AR (P)	–	↑ mtROS	–	–	↑ Frequency of apoptosis ↑ Caspase3 activity ↑ Bax ↑ Cyt‐C	–	–		28
**Wild‐type mice**												
Tamoxifen generated podocyte Drp1 null	–	–	↑ AR (P)	–	–	–	–	–	↔ Histology ↔ No. P/ glom area	↔ UACR	Diabetes increased Drp1, and genetically removal of Drp1 was attenuated kidney damage.	32
**db/db diabetic mice**												
Tamoxifen generated podocyte Drp1 null	–	↓ mt fragment	↑ AR (P)	↑	↓ mtROS	↑	↑	–	↓ mm ↓ Foot process effacement ↓ GBM thickness	↓ UACR		32
**db/db diabetic mice**												
Drp1^S600A^ knock in	–	↓ Drp1	↑ AR (P) ↑ FF (P)	–	↓ mtROS	–	–	–	↓ mm ↓ Foot process effacement ↓ GBM thickness ↔ WT1	↓ UACR	S600 was the important site of Drp1 which required for mitochondrial fission activation.	41
**Wild‐type mice**												
Hemizygous Hq mutation in AIFm1 gene	↔ OPA1‐L1 ↔ OPA1‐L2 ↑ MFN1 ↑ MFN2	↓ OPA1‐S1 ↓ OPA1‐S2 ↓ OPA1‐S3 ↓ mt No. ↔ Drp1 ↔ MFF	↑ AR (T)	–	↑ mtROS ↑ NOX4 expression	↑	–	↓ AIFm1 expression ↓ AIF protein	↑ IFTA ↑ GSI ↑ Glom Col IV ↑ Tubular Col IV ↑ Fn1	↑ UAE ↑ Urine KIM‐1 ↑ Urine NGAL ↓ Plasma Cystatin C ↑ CrCl	Partial AIF knockout gene did not have the effect on mitochondrial dynamics and function among diabetes mice.	42
**Hemizygous Hq mutation in AIFm1 gene mice**												
STZ‐induced diabetes mice	↔ MFN1 ↔ MFN2 ↔ OPA1	↔ Drp1 ↔ MFF	–	–	↑ mt3‐NT ↔ mtROS ↔ NOX4	↓	–	–	↑ GSI ↑ Glom Col IV ↑ Tubular Col IV ↔ Fn1	↑ UAE ↑ Urine KIM‐1 ↓ Plasma Cystatin C ↔ CrCl		42
**STZ‐induced diabetes mice**												
Hemizygous Hq mutation in AIFm1 gene	↔ MFN1 ↔ MFN2 ↔ OPA1	↔ Drp1 ↔ MFF	↓ AR (T)	–	↔ mt3‐NT ↔ mtROS ↔ NOX4	↔	–	–	↔ GSI ↔ Glom Col IV ↔ Tubular Col IV ↔ Fn1	↔ UAE ↔ Urine KIM‐1 ↔ Plasma Cystatin C ↔ CrCl		42

Abbreviations: AR, aspect ratio; ATP, adenosine triphosphate; Bax, Bcl‐2 associated X protein; Bcl‐2, B‐cell lymphoma 2; BUN, blood urea nitrogen; c‐IAP1, Cellular Inhibitor of Apoptosis Protein 1; c‐IAP2, Cellular Inhibitor of Apoptosis Protein 2; Col, collagen; CrCl, creatinine clearance; Cyt‐C, cytochrome C; Drp1, dynamic‐related protein 1; DsbA‐L, Disulphide‐bond A oxidoreductase‐like protein; DUSP1, Dual‐specificity protein phosphatase 1; FF, form factor; FIS1, fission 1 protein; Fn, fibronectin; Glom, glomerular; GSH, glutathione; GSI, glomerulosclerosis index; GSSG oxidized glutathione; H_2_O_2_, hydrogen peroxide; IFTA; interstitial fibrosis and tubular atrophy; IP, intraperitoneal; JNK, c‐Jun N‐terminal kinase; KIM‐1, kidney injury molecule‐1; LPO, lipid hydroperoxides; mALB; microalbuminuria; MFF, mitochondrial fission factor; MFN, mitofusin; mm, mesangial matrix; Mo, morphology; mt, mitochondria; mPTP, mitochondrial permeability transition pore; mRNA, messenger ribonucleic acid; 3‐NT, 3‐Nitrotyrosine; NAG, N‐acetyl‐beta‐D‐glucosaminidase; 8‐OHdG, 8‐Oxo‐2‐deoxyguanosine; OCR, oxygen consumption rate; OPA1, optic atrophy 1; P, podocyte cell; PGC‐1α, peroxisome proliferator‐activated receptor‐gamma coactivator 1‐alpha; Rap1b, Ras‐proximate 1b; ROCK1, Rho‐associated coiled coil‐containing protein kinase 1; ROS, reactive oxygen species; SCr, serum creatinine; SOD, superoxide dismutase; STZ, streptozotocin; T, tubular cell; TUNEL, terminal deoxynucleotidyl transferase dUTP nick end labelling; UACR, urine albumin creatinine ratio; UAE, urinary albumin excretion; UGGT, urinary gamma‐glutamyl transferase.

## 4 MITOCHONDRIAL DYNAMICS ALTERATIONS IN DIABETIC KIDNEY DISEASE: REPORTS FROM *CLINICAL* STUDIES

Currently, available clinical report on this topic is still scarce. The perturbation of mitochondrial dynamics among DKD patients compared with the non‐diabetic kidney disease group were consistent with the data reported from both *in vitro* and *in vivo* studies.^36^, ^42^, ^43^ Interestingly, there is evidence demonstrating the correlation between the mitochondrial dynamics and several clinical renal parameters.^36^, ^42^, ^43^ A positive correlation between mitochondrial AR and the degree of proteinuria,^43^ and a negative correlation of Rap1b expression and urine N‐Acetyl‐/β‐glucosaminidase (β‐NAG) level^36^ were found in DKD patients. Since most of these biomarkers for mitochondrial dynamics are not yet available in a clinical setting, the validation of those associations should be warranted in the larger clinical studies. A summary of findings from those *clinical* reports is shown in Table 3.

**TABLE 3:** Mitochondrial dynamics alterations in diabetic kidney disease: reports from *clinical* studies

Model	Baseline characteristics	Major findings						Histology	Renal parameters	Interpretation	Ref
		Mitochondrial dynamics		Mitochondrial function			Apoptosis				
		Fusion	Fission	mtMo	ΔΨm	Oxidative stress					
DKD biopsy proven (*n* = 31) vs. non‐DKD (*n* = 6)	SCr: 2.01 mg/dl vs.0.79 mg/dl Urine protein (g/L): 5.4 vs. 0.8	–	↑ mt fragment ↑ Drp1 ↑ AKAP1	↓ AR (P)	–	–	–	–	–	Increased mitochondrial fission and decreased aspect ratio were found in diabetic kidney disease patients.	43
DKD (*n* = 12) vs. non‐DM (*n* = 12)	Duration of diabetes: 10–15 years	–	↓ Rap1b	↓ AR (T)	–	–	–	↑ IFTA	↑ SCr ↑ Urine β‐NAG	Rap1b was decreased among diabetic patients and was negatively correlated with Urine β –NAG, and degree of interstitial fibrosis and tubular atrophy.	36
DKD (*n* = 15) vs. non‐DM (*n* = 2)	eGFR 40 vs. 70 Urine albumin (mg/L): 1044	–	–	–	–	–	↓ AIFM1 mRNA ↓ AIF positive (%cortical area) ↓ AIF positive (per tubule cross‐section)	–	–	AIF transcription and expression were decreased among diabetic kidney disease and was positively correlated with eGFR.	42

Abbreviations: AIF, apoptosis‐inducing factor; AIFM1, apoptosis‐inducing factor mitochondria‐associated 1; AKAP1, A‐kinase anchoring protein 1; AR, aspect ratio; Bax, Bcl‐2 associated X protein; Cyt‐C, cytochrome C; DKD, diabetic kidney disease; DM, diabetes mellitus; Drp1, dynamic‐related protein 1; eGFR, estimated glomerular filtration rate; FIS1, fission 1 protein; IFTA; interstitial fibrosis and tubular atrophy; MFN, mitofusin; Mo, morphology; mt, mitochondria; NAG, N‐acetyl‐beta‐D‐glucosaminidase; P, podocyte cell; Rap1b, Ras‐proximate 1b; SCr, serum creatinine; T, tubular cell.

## 5 PHARMACOLOGICAL INTERVENTIONS ON MITOCHONDRIAL DYNAMICS IN DKD: REPORTS FROM *IN VITRO* STUDIES

**FIGURE 3:**
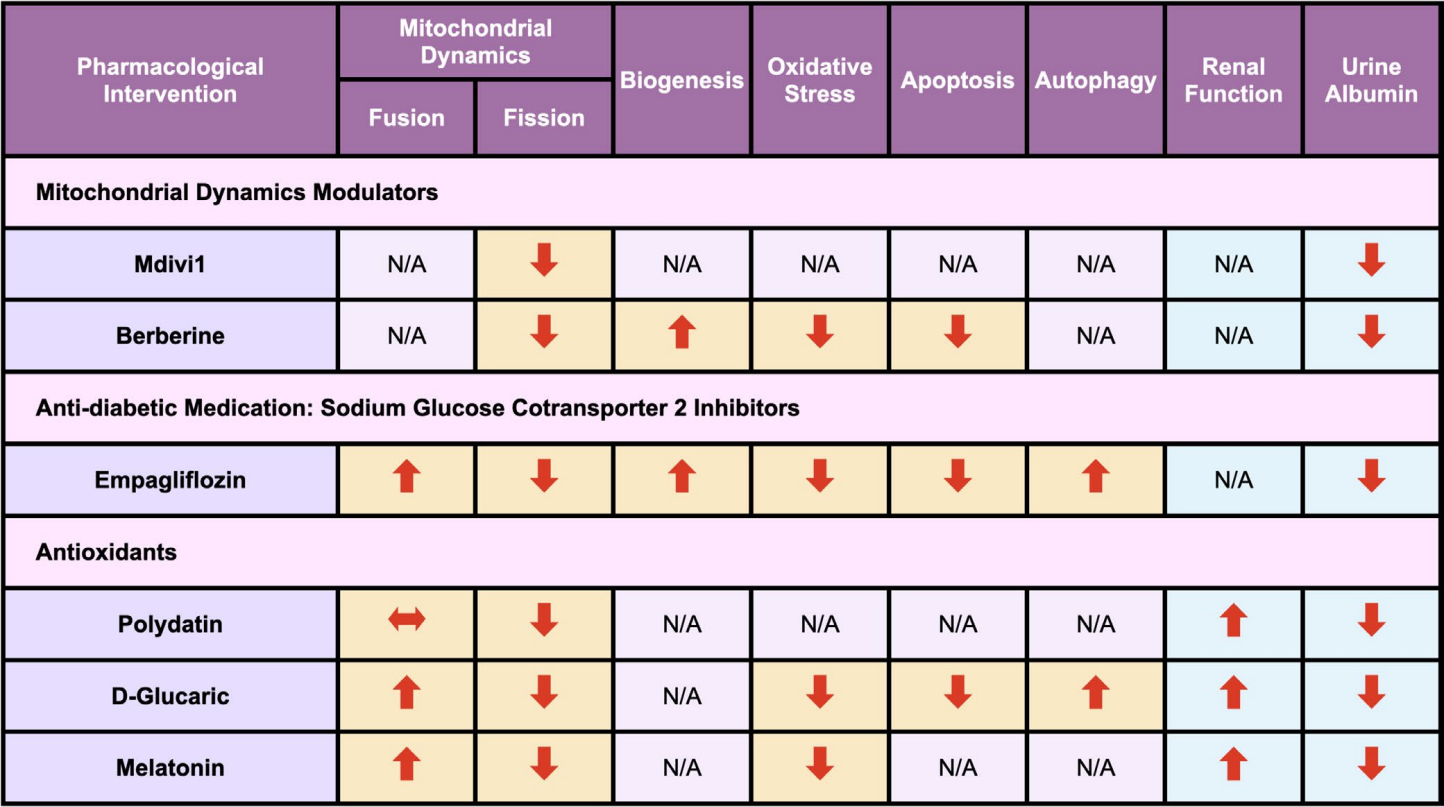
Pharmacological intervention on mitochondrial dynamics and their cooperative networks under diabetic milieu. According to the perturbation of mitochondrial dynamics and function in diabetic kidney disease, pharmacological interventions which had an impact on those process could be categorized into 3 main groups, including mitochondrial dynamics modulators, antidiabetic medication and antioxidants. Their effect on each process of mitochondrial dynamics, mitochondrial function, renal function and urine albumin were demonstrated in this figure. N/A, not available

According to the important role of mitochondrial dynamics on the pathogenesis of DKD, many emerging pharmacological interventions have been investigated. Accumulative evidence suggested that those medications could be categorized into 3 main groups, including mitochondrial dynamic modulators, antidiabetic medications and antioxidant therapies (Figure 3). A summary of the pharmacological interventions of DKD on the mitochondrial dynamics from the *in vitro* reports is shown in Table 4.

**TABLE 4:** Pharmacological interventions of diabetic kidney disease and their effects on mitochondrial dynamics: reports from *in vitro* studies

Intervention	Major findings										Renal parameters	Interpretation		Ref	
	Mitochondrial dynamics		Mitochondrial function					Apoptosis	Autophagy	Biogenesis					
	Fusion	Fission	mtMo	ΔΨm	Oxidative stress	ATP	OCR								
**Mitochondrial dynamic modulators**															
*Conditionally immortalized mouse podocytes*															
Pretreatment Mdivi1 + HG	–	↓ pDrp1	↑ AR (P)	↑	↓ mtROS	↑	–	↓ AnnexinV	–	–	–	Mdivi1, the Drp1 inhibitor, ameliorated the high glucose‐induced mitochondrial fission, oxidative stress, cellular apoptosis, and increased mitochondria aspect ratio.		32	
*Conditionally immortalized mouse podocyte (MPC‐5)*															
Berberine pretreatment + PA	–	↓ mRNA Drp1 ↓ pDrp1S616 ↓ FIS1 ↓ MFF ↓ Mid49	↑ AR (P)	↑	↓ mtROS ↓ MDA	↑	–	↓ AnnexinV ↓ Condensed chromatin ↓ Cleaved Caspase3 ↓ Bax ↓ Cyt‐C ↑ Bcl‐2	–	↑ mRNA PGC‐1α ↑ PGC‐1α ↑ mRNA TFAM ↑ mRNA NRF1 ↑ mRNA NRF2	↑ Nephrin ↑ Podocin ↑ CD2AP ↓ Desmin ↓ MMP‐9	Berberine ameliorated the effect of palmitic acid‐induced alteration of mitochondrial dynamics by decreasing the transcription of Drp1 and its protein receptors expression.		44	
Mdivi1 pretreatment + PA	–	↓ pDrp1S616 ↓ mRNA MFF	↑ AR (P)	↑	↓ mtROS ↓ MDA	↑	–	↓ AnnexinV ↓ Condensed chromatin ↓ Cleaved Caspase3 ↓ Bax ↓ Cyt‐C ↑ Bcl‐2	–	↑ PGC‐1α ↑ mRNA ND1	↑ Nephrin ↑ Podocin ↑ CD2AP ↓ Desmin ↓ MMP‐9				
Berberine pretreatment + lentivirus overexpressing Drp1 + PA vs. PA	–	↔ mt fragment ↔ pDrp1 ↔ Drp1	–	–	–	–	–	↔ AnnexinV ↔ Bcl‐2 ↔ Cleaved Caspase3 ↔ Bax ↔ Cyt‐C	–	–	↔ Nephrin ↔ Podocin ↔ CD2AP ↔ Desmin ↔ MMP‐9				
Berberine pretreatment + siDrp1 + PA vs. PA	–	↓ mt fragment ↓ pDrp1 ↓ Drp1	–	–	–	–	–	↓ AnnexinV ↑ Bcl‐2 ↓ Cleaved Caspase3 ↓ Bax ↓ Cyt‐C	–	–	↑ Nephrin ↑ Podocin ↑ CD2AP ↓ Desmin ↓ MMP‐9				
**Antidiabetic medication: sodium‐glucose cotransporter 2 inhibitors**															
*HKC‐8 cells, human renal proximal tubular cells*															
Empagliflozin + HG	↑ MFN1	↓ Drp1	–	↑	↓ mtROS	–	↑	↓ Bax/Bcl‐2 ratio ↓ Cyt‐C	↑ LC3‐II ↑ Beclin ↓ p62 ↓ mTORC1 complex ↓ Raptor protein ↑ ULK1	↑ p‐AMPKα	↓ Tim‐1	Empagliflozin improved mitochondrial dynamics by promoting mitochondrial fusion, and suppressing mitochondrial fission, concomitant with restoration of biogenesis and mitophagy.		53	
**Antioxidants**															
*MPC‐5*															
Polydatin + HG	–	↓ mt fragment ↓ Drp1 ↓ p‐616Drp1	↑ AR (P)	↑	↓ mtROS	↑	–	↓ Apoptotic cell ↓ Caspase3 activity ↓ Caspase9 activity ↓ Cyt‐C	–	–	↑ Nephrin ↑ Podocin	Protective effect of polydatin on podocyte under HG was mainly from its antioxidant property, independent on Drp1 action.		54	
*MPC‐5 transfected with lentivirus overexpress Drp1*															
Polydatin 25 mM + HG	–	–	–	–	–	–	–	↓ Apoptosis	–	–	↑ Nephrin ↑ Podocin			54	
*MPC‐5 transfected with Drp1‐siRNA*															
Polydatin 25 mM + HG	–	–	–	–	–	–	–	↔ Apoptosis ↔ Cleaved Caspase3	–	–	↔ Nephrin ↔ Podocin			54	
*Primary podocyte from KKAy mice*															
Polydatin 25 mM + HG	–	↓ Drp1	–	–	↓ mtROS	–	–	–	–	–	–				54
Polydatin 25 mM + HG vs. NAC + HG	–	↔ Drp1	–	–	↔ mtROS	–	–	–	–	–	–				
*HK‐2 cells (human) or LLC‐PK1 (porcine)*															
D‐glucaric acid, MIOX inhibitor + HG	↑ MFN2	↓ mt fragment ↓ Drp1	–	–	↓ mt ROS ↓ MIOX expression	–	–	↓Apoptotic cell ↓Bax ↓Cyt‐C	↑LC3 ↑Pink1 ↑Parkin ‐MFN2 interaction	–	–	D‐glucaric acid, a MIOX inhibitor, decreased the mitochondrial fission, ROS generation, and increased mitophagy, and mitochondrial fusion.		13	

Abbreviations: AMPKα, Adenosine monophosphate‐activated protein kinase alpha; AR, aspect ratio; ATP, adenosine triphosphate; Bax, Bcl‐2 associated X protein; Bcl‐2, B‐cell lymphoma 2; Cyt‐C, cytochrome C; Drp1, dynamic‐related protein 1; FIS1, fission 1 protein; HG, high glucose; LC3, light chain 3; MDA, malondialdehyde; Mdivi1, mitochondrial division inhibitor 1; mECs, microvascular endothelial cells; MFF, mitochondrial fission factor; MFN, mitofusin; Mid49, mitochondrial dynamics proteins of 49 kDa; Mid51 mitochondrial dynamics proteins of 51 kDa; MIOX, myo‐inositol oxygenase; MMP‐9, matrix metalloproteinase‐9; Mo, morphology; mt, mitochondria; mTORC1, mammalian target of rapamycin complex 1; mRNA, messenger ribonucleic acid; NRF, Nuclear Respiratory Factor; OCR, oxygen consumption rate; P, podocyte cell; PA, palmitic acid; PGC‐1α, peroxisome proliferator‐activated receptor‐gamma coactivator 1‐alpha; Pink1, PTEN‐induced putative kinase 1; ROCK1, Rho‐associated coiled coil‐containing protein kinase 1; ROS, reactive oxygen species; TFAM, Mitochondrial transcription factor A; Tim‐1, T‐cell immunoglobulin mucin receptor 1; ULK1, Unc‐51 Like Autophagy Activating Kinase 1.

### 5.1 Mitochondrial dynamic modulators

Since DRP1 and other co‐operated fission proteins are the main effector components in mitochondrial fission process that are upregulated in diabetic milieu, the mitochondrial dynamic modulators are investigated mainly to attenuate these substrates activities.^32^, ^44^ In conditionally immortalized mouse podocytes, Mdivi1, the pharmacological inhibitor of Drp1, exhibited the ability to suppress renal mitochondrial fission through decreasing the phosphorylated DRP1, leading to the recovery of mitochondrial functions, and the decrease in apoptosis under high glucose state.^32^ Similar results were showed in Berberine administration to the conditionally immortalized mouse podocytes under Palmitic acid (PA), the free fatty acid (FFA) that was increased in DM and involved in the perturbation of mitochondrial dynamics.^44^ With a different mechanism, Berberine decreased fission activity by directly inhibited DRP1 transcription, concurrent with the downregulation of the expression of FIS1, MFF, mitochondrial dynamics proteins of 49 kDa (Mid49). Interestingly, Berberine also improved mitochondrial biogenesis and the increase of satisfactory marker of renal parameters, including Nephrin and Podocin.^44^ All of these findings suggested that mitochondrial dynamic modulators could reverse the effect of high glucose on renal mitochondrial dynamics, which might provide the benefits on the DKD. A summary of the effects of the mitochondrial dynamic modulators from the *in vitro* reports is shown in Table 4.

### 5.2 Antidiabetic drug: Sodium‐glucose cotransporter 2 inhibitors

Sodium‐glucose cotransporter 2 inhibitors (SGLT‐2i) is a new group of pharmacological intervention that has been shown to slow the DKD progression.^45^, ^46^, ^47^ Although SGLT‐2i is categorized to be one of the glucose‐lowering agents, their beneficial effects are proven to be beyond the glycaemic control.^48^ Not only the result of recovering tubulo‐glomerular feedback,^49^ lowering blood pressure^50^ and weight reduction^51^ that can explain the underlying reno‐protective effect but also the normalization of mitochondrial dysregulation that play a crucial role.^52^ Nevertheless, the mechanistic evaluation on those mitochondrial dynamics and functions was not apparently elucidated.

Administration of empagliflozin, one of the SGLT‐2i, to human renal proximal tubular cells (hRPTCs) under a diabetic milieu could increase mitochondrial fusion and reduce mitochondrial fission.^53^ Furthermore, it also improved mitochondrial function, autophagy and biogenesis, concurrently with an attenuation of apoptosis and the tubular injury. These favourable outcomes were comparable to the SGLT2‐silencing hRPTCs under high glucose environment.^53^ Taken together, this study emphasized the potential additional reno‐protective benefit of SGLT‐2i on DKD and supported that mitochondrial abnormality was one of the underlying pathogenesis, leading to the kidney progression and ESRD among diabetes patients. A summary of the effects of the antidiabetic drug from the *in vitro* reports is shown in Table 4.

### 5.3 Antioxidant therapies: Polydatin and D‐glucaric acid

Several antioxidants including Polydatin and D‐glucaric acid have been investigated under the diabetic milieu. A summary of those reports regarding the use of antioxidants in *in vitro* studies is shown in Table 4.

Polydatin (PD) is resveratrol glycoside, which is extracted from the radix of Polygonum cuspidatum.^54^ Current evidence disclosed that PD has the anti‐oxidative property.^55^ In conditionally mouse podocytes (MPC‐5) under high glucose state, PD attenuated mitochondrial fission as indicated by decreased phosphorylation of DRP1 at Serine‐616.^54^ In addition, PD improved the mitochondrial AR and bioenergetic function, concurrently with a reduction in the ROS and apoptosis. Interestingly, both transfected DRP1 silencing RNA and administered of PD to mouse podocyte cell line exerted similar efficacy in attenuating apoptosis.^54^ Similarly, administration of D‐glucaric acid, which decreased ROS through inhibiting the MIOX, to the renal proximal tubular cell of human (HK‐2) and porcine (LLC‐PK1) under diabetic ambience was shown to attenuate renal mitochondrial fission, oxidative stress, accompanied with the recovery of renal mitochondrial fusion and autophagy through stimulating PINK1 facilitated Parkin‐MFN2 interaction.^13^ These findings indicated that oxidative stress has a role in the alteration of mitochondrial dynamics in DKD.

## 6 PHARMACOLOGICAL INTERVENTIONS OF MITOCHONDRIAL DYNAMICS IN DKD: REPORTS FROM *IN VIVO* STUDIES

*In vivo* studies provided the important information of the pharmacological interventions on the laboratory parameters. Similar with the *in vitro* reports, therapeutic drugs that aimed to restore the balance of mitochondrial dynamics and their co‐operate networks from previous *in vivo* reports could be mainly classified into 3 categories (Figure 3). A summary of the pharmacological interventions on the mitochondrial dynamics of DKD from the *in vivo* reports is shown in Table 5.

**TABLE 5:** Pharmacological interventions of diabetic kidney disease and their effects on mitochondrial dynamics: reports from *in vivo* studies

Method (Drug/dose/route/duration)	Major findings									Histology		Renal parameter	Interpretation	Ref
	Mitochondrial dynamics		Mitochondrial function				Apoptosis	Autophagy	Biogenesis					
	Fusion	Fission	mtMo	ΔΨm	Oxidative stress	ATP								
**Mitochondrial dynamic modulators**														
*db/db mice*														
Mdivi1/50 mg/kg/IP EOD/8 weeks	–	↓ mt fragment	↑ AR (P)	–	–	–	–	–	–		↓ mm ↑ No. P /glom area ↓ GBM thickness	↓ UACR	Mdivi1 reversed the effect of diabetes‐associated mitochondrial dysfunction, clinical parameter, and the kidney histology in diabetic mice.	32
*db/db mice*														
Berberine/300 mg/kg/d/gavage/8 weeks	–	↓ mt pDrp1 (S616) ↑ Cyto pDrp1 (S616) ↓ Drp1 mRNA level ↓ Mid 49 ↓ Mid51 ↓ MFF ↓ FIS1	↑ AR (P)	–	↓ mtROS	–	↑ Bcl‐2 ↓ Bax ↓ Cleaved Caspase3 ↓ Cyt‐C	–	↑ PGC‐1α ↑ NRF1 ↑ NRF2 ↑ TFAM ↑ ND1		↑ No. of P ↓ Foot process effacement ↓ mm ↓ GBM thickness	↓ UACR ↓ MMP‐9 ↓ Desmin ↑ Nephrin ↑ Podocin	Berberine reversed the effect of diabetes on mitochondrial dynamics by inhibiting the mitochondrial fission, ameliorating oxidative stress, and promoting mitochondrial biogenesis.	44
**Antidiabetic medication: sodium‐glucose cotransporter 2 inhibitors**														
*STZ‐induced diabetes mice*														
Empagliflozin 300 mg/kg/ PO 24 h. interval/12 weeks	↑ MFN1	↓ mt fragment ↓ Drp1	–	–	↓ 8‐OHdG	–	↓ Bax/ Bcl‐2 ratio ↓ Cyt‐C ↓ TUNEL	↑ LC3‐II ↑ Beclin ↓ p62 ↓ mTOR ↓ Raptor ↑ ULK1	↑ p‐AMPKα ↑ AMPKα		↓ Tubular injury ↓ Fibrosis score	↓ UACR ↓ Kidney to BW ratio	Empagliflozin improved diabetic kidney disease by decreasing mitochondrial fission, and oxidative stress, promoting mitochondrial fusion, and biogenesis, and attenuating impaired autophagic process.	53
**Antioxidants**														
*STZ‐induced diabetes mice*														
1% or 2% glucarate added to dietary chow 1 week after STZ for 8 weeks	↑ MFN2	↓ mt fragment ↓ Drp1 ↓ FIS1	↑ AR (T)	–	↓ DHE fluorescence intensity ↓ MIOX activity	–	↓ Bax ↓ Cyt‐C ↓ TUNEL	↑ LC3 ↑ Atg5 ↑ Pink1	–		↓ Tubular disruption ↓ Glom hypertrophy ↓ mm ↓ Glom damage score (2% glucarate) ↓ Tubular damage score	↓ SCr ↓ UACR	D‐glucarate improved diabetic kidney disease, mainly by ameliorating tubular injury, through reducing oxidative stress and mitochondrial dynamic imbalance.	13
*Diabetic fatty rats*														
Melatonin/10 mg/kg/d/PO/17 weeks	↑ MFN2 ↑ OPA1	↓ Drp1	–	–	↑ SOD ↓ Nitrites	–	–	–	–		–	↑ CrCl ↓ UACR	Melatonin improved kidney injury in diabetic fatty rats via increasing mitochondrial fusion and reducing fission.	67
*High‐fat diet‐fed KKAy mice*														
Polydatin/100 mg/kg/d/8 weeks	–	↓ Drp1 ↓ p‐616Drp1	–	–	–	–	–	–	–		↓ mm ↓ Foot process effacement ↑ Slit width	↓ 24‐hr Urine protein ↓ Urine albumin ↓ BUN ↓ SCr ↑ Nephrin ↑ Podocin	Polydatin improved diabetic kidney disease through repressing mitochondrial fission.	54

Abbreviations: AMPKα, Adenosine monophosphate‐activated protein kinase alpha; APN, adiponectin; AR, aspect ratio; Atg5, autophagy‐Related Gene 5; ATP, adenosine triphosphate; Bax, Bcl‐2 associated X protein; Bcl‐2, B‐cell lymphoma 2; BUN, blood urea nitrogen; BW, body weight; CrCl, creatinine clearance; Cyt‐C, cytochrome C; DHE, Dihydroethidium; Drp1; dynamin‐related protein 1; FFA, free fatty acid; Glom, glomerular; FIS1, fission 1 protein; GBM, glomerular basement membrane; IF, interstitial fibrosis; LC3, light chain 3; Mdivi1, mitochondrial division inhibitor 1; MFF, mitochondrial fission factor; MFN, mitofusin; Mid49, mitochondrial dynamics proteins of 49 kDa; Mid51 mitochondrial dynamics proteins of 51 kDa; mm, mesangial matrix; MIOX, myo‐inositol oxygenase; MMP‐9, matrix metalloproteinase‐9; Mo, morphology; mRNA, messenger ribonucleic acid; mt, mitochondria; mTOR, mammalian target of rapamycin; NAG, N‐acetyl‐beta‐D‐glucosaminidase; NRF, Nuclear Respiratory Factor; 8‐OHdG, 8‐Oxo‐2‐deoxyguanosine; OPA1, optic atrophy 1; P, podocyte cell; PGC‐1α, peroxisome proliferator‐activated receptor‐gamma coactivator 1‐alpha; Pink1, PTEN‐induced putative kinase 1; PO, per oral; RhoA‐GTP, Ras homolog family member A Guanosine‐5′‐triphosphate; ROCK1, Rho‐associated coiled coil‐containing protein kinase 1; SCr, serum creatinine; SOD, superoxide dismutase; T, tubular cell; TFAM, Mitochondrial transcription factor A; TUNEL, terminal deoxynucleotidyl transferase dUTP nick end labelling; UACR, urine albumin creatinine ratio; UAE, urinary albumin excretion; ULK1, Unc‐51 Like Autophagy Activating Kinase 1.

### 6.1 Mitochondrial dynamic modulators

Mdivi1 and Berberine were demonstrated a promising benefit on both kidney histology and the surrogate marker of renal outcome like urinary albumin excretion among genetically diabetic mice model.^32^, ^44^ According to the same pharmacological action as DRP1 activity inhibitors, both remedies could attenuate the renal mitochondrial fission and normalized the mitochondrial AR. Berberine was also shown to reduce mitochondrial oxidative stress, apoptosis and biogenesis.^44^

### 6.2 Antidiabetic drug: Sodium‐glucose cotransporter 2 inhibitors

Despite its benefits reported in many recent clinical studies of DKD and chronic kidney disease,^46^, ^47^, ^56^, ^57^ the mechanistic insight regarding its effects on the mitochondrial networking is still limited. In streptozotocin (STZ)‐induced diabetic mice, empagliflozin attenuated renal mitochondrial fission and increased renal mitochondrial fusion, when compared to the diabetic control.^53^ In addition, oxidative stress and apoptosis were decreased, while the autophagy and biogenesis were recovered. The clinical parameters including the renal histology and the degree of urinary albumin were also improved.^53^

According to the pathogenesis of the DKD, emerging evidence demonstrated that the SGLT‐2i could modulate all of those pathways, including metabolic,^58^, ^59^, ^60^ haemodynamics^61^ and the latest concept of the perturbation of mitochondrial dynamics.^53^ These crucial issues instigated the hypothesis that SGLT‐2i could halt the progression, and attenuate the pathology of kidneys in DKD.

The benefit of SGLT‐2i in CKD from other aetiologies is still limited. However, data from the pre‐specified analysis of DAPA‐CKD trial which is the study aimed to evaluate the efficacy of dapagliflozin, one of the SGLT‐2i, on the primary kidney outcome demonstrated that dapagliflozin also had benefit in participants with non‐diabetes CKD, specifically, IgA nephropathy.^47^ Nonetheless, the precise mechanisms related to these outcomes were not clearly defined, and therefore, whether this benefit is also possibly due to the improvement of the renal mitochondrial dynamics as demonstrated in DKD models are to be determined. Hence, future studies are needed to explore the precise mechanisms and to warrant its use in DKD and CKD from other aetiologies.

### 6.3 Antioxidant therapies

#### 6.3.1 Polydatin and D‐glucaric

Oxidative stress is found to involved in the development of DKD through directly affect the kidney cells, and also worsen the alteration of mitochondrial dynamics.^19^ In diabetic mice, both Polydatin and D‐glucaric could attenuate renal mitochondrial fission, abnormal renal histology, simultaneously with improved renal function and urinary albumin excretion.^13^, ^54^ D‐glucaric also demonstrated the efficacy in promoting mitochondrial fusion, autophagy and reducing apoptosis. These findings indicated the important role of oxidative stress in DKD.

Despite those benefits of antioxidants have been demonstrated, the role of anti‐oxidative therapy on DKD is still controversial. Although the clinical studies of Bardoxolone methyl, an antioxidant inflammatory modulator, demonstrated that it could improve eGFR,^62^ it was reported to be associated with the increased blood pressure and albuminuria, and the increased incidence of the cardiovascular events.^63^ In addition, the beneficial effect on the development of ESRD was not determined. Future studies regarding the use of novel antioxidant therapy should be further investigated for their efficacies and safety profiles.

#### 6.3.2 Melatonin

Melatonin is a pleiotropic hormone which regulates circadian rhythm and preserves mitochondrial stability through antioxidant and anti‐inflammatory properties.^64^ Previous studies demonstrated the reno‐protective effects of melatonin under diabetes and obesity‐induced nephropathy.^65^, ^66^ These satisfactory results were linked to the modulation on renal mitochondrial dynamics by increasing of MFN2 among obesity mouse model.^66^ Melatonin could also increase the expression of mitochondrial fusion proteins, including MFN2 and OPA1, whereas it suppressed the mitochondrial fission process in diabetic fatty rat model.^67^ In addition, it promoted the superoxide dismutase (SOD), an anti‐oxidative enzyme, concurrent with decreased oxidative stress.^67^ These findings were associated with the improvement of creatinine clearance and albuminuria in diabetic fatty rat model.^67^ Although whether the beneficial effects of melatonin on DKD were directly through modulating the renal mitochondrial dynamics or the consequences of the attenuation of oxidative stress was not totally elucidated, the reno‐protective effect of melatonin was suggested. Taken together, melatonin could reverse the perturbation of renal mitochondrial dynamics and functions, simultaneously with the improved clinical parameters among *in vivo* DKD models.

## 7 CONCLUSION

Alteration of mitochondrial dynamics towards fission is disclosed in DKD. This contributes to the excessive oxidative stress generation, cellular apoptosis, bioenergetic dysfunction, and impaired autophagy and biogenesis. Unfortunately, those changes conversely insult the mitochondrial dynamics, leading to the worsening of renal structures and functions. The underlying regulatory proteins and signalling which govern on mitochondrial dynamics and their complex interconnected networks have been shown to play an important role in DKD. Interestingly, restoring those mitochondrial dynamics leads to the improvement of the renal parameters. However, the evidence of those benefits among human is still limited. Future clinical studies with large population are needed to disclose the mystery regarding the roles of mitochondrial dynamics in DKD patients. In addition, pharmacological studies of mitochondrial modulators and SGLT‐2i as well as other novel remedies are encouraged to prove their advantages on the DKD progression and the anticipation of ESRD risk elimination among DM patients.

## CONFLICT OF INTEREST

The authors declare that there is no conflict of interest to declare.

## AUTHOR CONTRIBUTIONS

**Phoom Narongkiatikhun:** Conceptualization (lead); Formal analysis (lead); Methodology (lead); Writing – original draft (lead). **Siriporn Chattipakorn:** Conceptualization (supporting); Funding acquisition (lead); Methodology (supporting); Supervision (supporting); Writing – review & editing (supporting). **Nipon Chattipakorn:** Conceptualization (supporting); Funding acquisition (lead); Methodology (supporting); Project administration (lead); Supervision (lead); Writing – review & editing (lead).
